# Prevalence and molecular characterization of enteric zoonotic pathogens in zoo-housed mammals in Korea

**DOI:** 10.1016/j.onehlt.2026.101568

**Published:** 2026-09-23

**Authors:** Hyo-Min Woo, In Jung Jung, Beoul Kim, You-Jeong Lee, Jae-Woo Choi, Insu Choi, Garam Kim, Weon-Hwa Jheong, Byoungcheun Lee, Kyoo-Tae Kim, Min-Goo Seo

**Affiliations:** a College of Veterinary Medicine & Institute for Veterinary Biomedical Science*,* Kyungpook National University, 80 Daehak-ro, Buk-gu*,* Daegu 41566*,* Republic of Korea; b Wildlife Disease Response Team, National Institute of Wildlife Disease Control and Prevention (NIWDC)*,* Ministry of Climate, Energy and Environment (MCEE), 1 Songam-gil*,* Gwangju 62407*,* Republic of Korea

**Keywords:** Zoo-housed mammals, E*nterocytozoon bieneusi*, *Giardia intestinalis*, *Cryptosporidium parvum*, Enteric pathogens, One health

## Abstract

Zoos represent important One Health interfaces where captive wildlife, animal caretakers, visitors, and shared environments are closely connected. This study investigated intestinal parasites and selected zoonotic pathogens in zoo-housed mammals in Korea and characterized the detected enteric pathogens. A total of 367 individual fecal samples were collected from mammals in nine zoos between September and November 2025. Microscopic examination and molecular assays targeting *Giardia intestinalis, Cryptosporidium* spp., *Enterocytozoon bieneusi, Toxoplasma gondii, Coxiella burnetii, Blastocystis* sp., and severe fever with thrombocytopenia syndrome virus (SFTSV) were performed. The overall positivity rate was 6.0%. Microscopy detected coccidian oocysts, ascarid-type eggs, hookworm-like eggs, and trichostrongyle-type eggs. Molecular analysis identified *E. bieneusi* as the most frequently detected pathogen, followed by *G. intestinalis* and *C. parvum*, whereas *T. gondii, C. burnetii, Blastocystis* sp., and SFTSV were not detected. Phylogenetic analysis showed that *G. intestinalis* sequences clustered with assemblage B and *C. parvum* sequences clustered within the *C. parvum* clade. ITS sequence and phylogenetic analyses further identified *E. bieneusi* genotype Type IV within Group 1 and a distinct genotype provisionally designated SealKB1 within Group 11. To the best of our knowledge, this study provides the first molecular evidence of *G. intestinalis* in African crested porcupines and *E. bieneusi* in Eurasian lynxes and harbor seals in Korea. These findings provide baseline data for zoo-based pathogen surveillance and support continued One Health monitoring in zoological institutions.

## 1 Introduction

One Health emphasizes the interconnection among human, animal, and ecosystem health [1]. Zoological institutions represent a unique One Health interface where captive wildlife, animal caretakers, veterinarians, visitors, and shared environments are closely connected [2]. In such settings, diverse animal species are maintained in confined spaces, and pathogens shed through feces may contaminate enclosures, water sources, feeding areas, bedding materials, and cleaning equipment. Therefore, zoo-based pathogen surveillance can provide useful information for animal health management, occupational health, environmental hygiene, and public health risk assessment.

Intestinal parasites and enteric zoonotic pathogens are particularly relevant in captive wildlife because many are transmitted by the fecal–oral route and can persist in contaminated environments. *Giardia intestinalis*, *Cryptosporidium* spp., and *Enterocytozoon bieneusi* have been detected in humans and diverse animal hosts, including wildlife and captive animals [3], [4], [5], [6], [7], [8], [9], [10]. These pathogens include species, assemblages, and genotypes with zoonotic potential, and their occurrence in zoo-housed animals may indicate possible environmental contamination and repeated exposure of susceptible hosts [6], [8], [9], [10].

In addition to these enteric pathogens, other zoonotic agents of public and veterinary health importance, including *Coxiella burnetii, Toxoplasma gondii, Blastocystis* sp., and severe fever with thrombocytopenia syndrome virus (SFTSV), may be relevant to wildlife surveillance in Korea [11], [12], [13], [14], [15]. These pathogens were selected based on their zoonotic relevance, previous detection in wildlife or captive animals, potential association with animal-derived environmental contamination, and public or veterinary health importance.

In Korea, studies on intestinal parasites and zoonotic pathogens have been conducted in wildlife, companion animals, and selected zoo animals [16], [17], [18], [19], [20], [21]. However, multi-institutional surveillance data on intestinal parasites and selected zoonotic pathogens in zoo-housed mammals remain limited. In particular, few studies have combined microscopic examination, molecular detection, and sequence-based characterization to evaluate pathogen occurrence across diverse captive mammalian taxa. This lack of baseline information limits the understanding of pathogen distribution in zoo environments and the development of evidence-based surveillance strategies.

Therefore, this study aimed to investigate intestinal parasites and selected zoonotic pathogens in fecal samples from zoo-housed mammals in Korea, characterize the molecular features of the detected enteric pathogens, and evaluate their implications for One Health-based surveillance in zoological institutions.

## 2 Materials and methods

### 2.1 Ethical approval

All procedures related to the use of fecal samples were reviewed and approved by the Institutional Animal Care and Use Committee of Kyungpook National University (approval no. 2025–0902). Fecal samples were non-invasively collected by veterinarians at the participating zoos as part of routine animal health management and were provided for this study with permission from each institution. No animals were handled, restrained, or experimentally manipulated by the researchers for the purpose of this study.

### 2.2 Collection of fecal samples

Fecal samples were collected from zoo-housed mammals in nine zoos in the Republic of Korea between September and November 2025. A total of 367 individual fecal samples were obtained from mammals representing 13 orders and 38 families. Freshly voided feces were collected immediately after defecation whenever possible, placed in sterile containers, transported under refrigerated conditions, and processed within 48 h of collection. The distribution of samples by taxonomic group is summarized in Table 1, and detailed species-level and zoo-specific sample distributions are provided in Supplementary Table S2.

**Table 1:** Distribution of intestinal parasites and selected zoonotic pathogens detected by microscopic and molecular examination in zoo-housed mammals.

Order	Family	No. samples tested	No. positive samples by microscopic examination (%)				No. positive samples by molecular examination (%)							Total
			Parasites				Bacterial pathogen	Protozoan and microsporidian pathogens					Viral pathogen	
			Ascarid-type eggs	Coccidian oocysts	Hookworm-like eggs	Trichostrongyle-type eggs	*Coxiella burnetii*	*Toxoplasma gondii*	*Giardia intestinalis*	*Cryptosporidium* spp.	*Enterocytozoon bieneusi*	*Blastocystis* sp.	SFTSV	
Artiodactyla	Bovidae	35	0	2 (5.7, CI: 0.7–19.2)	0	0	0	0	0	0	0	0	0	2 (5.7, CI: 0–13.4)
	Camelidae	6	0	0	0	0	0	0	0	0	0	0	0	0
	Cervidae	16	0	0	0	1 (6.3, CI: 0.2–30.2)	0	0	0	0	0	0	0	1 (6.3, CI: 0–18.1)
	Giraffidae	5	0	0	0	0	0	0	0	0	0	0	0	0
	Hippopotamidae	3	0	0	0	0	0	0	0	0	0	0	0	0
	Suidae	1	0	0	0	0	0	0	0	0	0	0	0	0
Carnivora	Canidae	39	0	0	0	0	0	0	0	0	0	0	0	0
	Felidae	30	2 (6.7, CI: 0.8–22.1)	0	1 (3.3, CI: 0.1–17.2)	0	0	0	0	0	1 (3.3, CI: 0.1–17.2)	0	0	4 (13.3, CI: 3.8–30.7)
	Herpestidae	9	0	0	0	0	0	0	0	0	0	0	0	0
	Hyaenidae	4	0	0	0	0	0	0	0	0	0	0	0	0
	Mustelidae	12	0	0	0	0	0	0	0	0	0	0	0	0
	Otariidae	12	0	0	0	0	0	0	0	0	0	0	0	0
	Phocidae	18	0	0	0	0	0	0	0	0	6 (33.3, CI: 13.3–59.0)	0	0	6 (33.3, CI: 13.3–59.0)
	Procyonidae	23	0	0	0	0	0	0	0	0	0	0	0	0
	Ursidae	18	0	0	0	0	0	0	0	0	1 (5.6, CI: 0.1–27.3)	0	0	1 (5.6, CI: 0.1–27.3)
Cingulata	Chlamyphoridae	2	0	0	0	0	0	0	0	0	0	0	0	0
Diprotodontia	Macropodidae	7	0	0	0	0	0	0	0	0	0	0	0	0
Hyracoidea	Procaviidae	4	0	0	0	0	0	0	0	0	0	0	0	0
Lagomorpha	Leporidae	8	0	0	0	0	0	0	0	0	0	0	0	0
Monotremata	Tachyglossidae	2	0	0	0	0	0	0	0	0	0	0	0	0
Perissodactyla	Equidae	15	0	0	0	0	0	0	0	0	0	0	0	0
	Rhinocerotidae	6	0	0	0	0	0	0	0	0	0	0	0	0
	Tapiridae	2	0	0	0	0	0	0	0	0	0	0	0	0
Pilosa	Choloepodidae	2	0	0	0	0	0	0	0	0	0	0	0	0
	Myrmecophagidae	2	0	0	0	0	0	0	0	0	0	0	0	0
Primates	Atelidae	2	0	0	0	0	0	0	0	0	0	0	0	0
	Callitrichidae	11	0	0	0	0	0	0	0	0	0	0	0	0
	Cebidae	10	0	0	0	0	0	0	0	0	0	0	0	0
	Cercopithecidae	24	0	1 (4.2, CI: 0.1–21.1)	0	0	0	0	0	2 (8.3, CI: 1.0–27.0)	1 (4.2, CI: 0.1–21.1)	0	0	4 (16.7, CI: 4.7–37.4)
	Hominidae	1	0	0	0	0	0	0	0	0	0	0	0	0
	Hylobatidae	8	0	0	0	0	0	0	0	0	0	0	0	0
	Lemuridae	1	0	0	0	0	0	0	0	0	0	0	0	0
	Lorisidae	3	0	0	0	0	0	0	0	0	0	0	0	0
Proboscidea	Elephantidae	5	0	1 (20.0, CI: 0.5–71.6)	0	0	0	0	0	0	0	0	0	1 (20.0, CI: 0.5–71.6)
Rodentia	Caviidae	5	0	0	0	0	0	0	0	0	0	0	0	0
	Hystricidae	4	0	0	0	0	0	0	3 (75.0, CI: 19.4–99.4)	0	0	0	0	3 (75.0, CI: 19.4–99.4)
	Sciuridae	11	0	0	0	0	0	0	0	0	0	0	0	0
Tubulidentata	Orycteropodidae	1	0	0	0	0	0	0	0	0	0	0	0	0
**Total**		367	2 (0.5, CI: 0.1–2.0)	4 (1.1, CI: 0.3–2.8)	1 (0.3, CI: 0.01–1.5)	1 (0.3, CI: 0.01–1.5)	0	0	3 (0.8, CI: 0.2–2.4)	2 (0.5, CI: 0.1–2.0)	9 (2.5, CI: 1.1–4.6)	0	0	22 (6.0, CI: 3.8–8.9)

### 2.3 Stool examination using the saturated sodium nitrate flotation technique

All fecal samples were examined for intestinal parasites using a saturated sodium nitrate flotation technique according to standard veterinary parasitological procedures [22]. The examination targeted helminth eggs, including ascarid-type, hookworm-like, and trichostrongyle-type eggs, as well as protozoan oocysts, including coccidian oocysts. Parasite stages were identified by light microscopy based on their morphological characteristics.

### 2.4 Nucleic acid extraction and molecular detection

Total nucleic acids were extracted from approximately 250 mg of each fecal sample using the Beniprep® Soil/Fecal DNA Extraction Kit (inVIRUSTECH, Gwangju, Republic of Korea) with a manufacturer-advised modified protocol incorporating an RPB buffer (the binding buffer used in the Clear-S™ Total RNA Extraction Kit; supplied separately for RNA recovery by inVIRUSTECH). Briefly, fecal material was homogenized in 600 μL of HBS lysis buffer supplemented with 10 μL of β-mercaptoethanol using bead beating at 3500 rpm for three 30-s cycles separated by 10-s intervals. The 65 °C heating step included in the standard DNA extraction protocol was omitted in accordance with the manufacturer's technical recommendation. After centrifugation at 13,000 rpm for 3 min at room temperature, approximately 450 μL of the supernatant was transferred to a new tube, mixed with 120 μL of CPS buffer, incubated at 4 °C for 5 min, and centrifuged at 13,000 rpm for 3 min at 4 °C. Approximately 500 μL of the clarified supernatant was then mixed with 1100 μL of RPB buffer supplied by the manufacturer and loaded onto the DNA binding column in two sequential 800-μL aliquots. The column was washed sequentially with 750 μL of WA-S buffer and 750 μL of Wash buffer and centrifuged for an additional 2 min to remove residual ethanol. Total nucleic acids were eluted with 150 μL of EB buffer prewarmed to 37 °C following a 5-min incubation at room temperature and stored at −70 °C until molecular analysis. This modified extraction procedure was used to prepare nucleic acid eluates for both DNA-target PCR assays and SFTSV reverse transcription PCR.

Molecular screening was performed for seven selected zoonotic pathogens: *C. burnetii, T. gondii, G. intestinalis*, *Cryptosporidium* spp., *E. bieneusi, Blastocystis* sp., and SFTSV. *C. burnetii* was detected by conventional PCR targeting the IS1111 transposase gene [23]. *T. gondii, G. intestinalis, Cryptosporidium* spp., and *E. bieneusi* were detected by nested PCR targeting the B1 gene, 18S rRNA gene, 18S rRNA gene, and internal transcribed spacer region, respectively [24], [25], [26], [27].

*Blastocystis* sp. was detected by PCR targeting the 18S rRNA gene [28], and SFTSV was detected by nested reverse transcription PCR [29]. Details of primer sets, target genes, amplicon sizes, assay types, and references are provided in Supplementary Table S1. Positive and negative controls were included in each PCR run, and nested PCR assays were performed in physically separated pre- and post-amplification areas to minimize carry-over contamination.

### 2.5 DNA sequencing and phylogenetic analysis

PCR-positive amplicons of the expected size were submitted to Macrogen (Seoul, Republic of Korea) for bidirectional Sanger sequencing. The obtained sequences were edited and assembled using BioEdit version 7.2.5 and compared with reference sequences available in the NCBI GenBank database using BLASTn. Multiple sequence alignments were performed using CLUSTAL Omega. Phylogenetic analyses were conducted using MEGA version 6.0 [30]. Evolutionary distances were calculated using the Kimura two-parameter model [31], and phylogenetic trees were constructed using the maximum likelihood method with 1000 bootstrap replicates. The sequences generated in this study were deposited in the GenBank database under the following accession numbers: *G. intestinalis* 18S rRNA, PX927595–PX927597; *C. parvum* 18S rRNA, PX904839–PX904840; and *E. bieneusi* ITS, PX927601–PX927603 for genotype Type IV and PX927604–PX927609 for the provisionally designated genotype SealKB1.

For *E. bieneusi*, genotype designation was based on the complete 243-bp ITS region according to the established nomenclature system of Santín and Fayer [32]. Study sequences were compared with previously described genotype sequences available in GenBank. Sequences showing 100% identity across the complete ITS region were assigned the corresponding established genotype name, whereas unique ITS sequences without an identical match to a previously described genotype were provisionally designated as novel genotypes and subsequently assigned to phylogenetic groups.

### 2.6 Statistical analysis

Statistical analyses were performed using GraphPad Prism version 5.04 (GraphPad Software Inc., La Jolla, CA, USA). Positivity rates were calculated as the number of positive samples divided by the number of tested samples, and exact binomial 95% confidence intervals (CIs) were calculated using the Clopper–Pearson method. Because sample sizes were highly uneven among host taxonomic groups and several groups contained only a small number of animals, subgroup-specific positivity rates were treated as descriptive estimates, and no formal statistical comparisons among taxonomic groups were performed.

## 3 Results

### 3.1 Overall positivity by microscopic and molecular examinations

A total of 367 individual fecal samples were analyzed. Overall, 22 samples were positive by either microscopic examination or molecular analysis, corresponding to a positivity rate of 6.0% (22/367; 95% CI: 3.8–8.9). Microscopic examination detected intestinal parasites in 8 samples (2.2%, 8/367; 95% CI: 0.9–4.2), whereas molecular analysis detected selected enteric pathogens in 14 samples (3.8%, 14/367, 95% CI: 2.1–6.3). Positivity was detected across several taxonomic groups; however, subgroup-specific estimates were considered descriptive because of the highly uneven sample sizes among taxonomic groups.

### 3.2 Microscopic detection of intestinal parasites

Microscopic examination detected intestinal parasites in 8 of 367 fecal samples (2.2%; 95% CI: 0.9–4.2). Coccidian oocysts were the most frequently detected parasitic stage (1.1%, 4/367; 95% CI: 0.3–2.8), followed by ascarid-type eggs (0.5%, 2/367; 95% CI: 0.1–2.0), hookworm-like eggs (0.3%, 1/367; 95% CI: 0.01–1.5), and trichostrongyle-type eggs (0.3%, 1/367; 95% CI: 0.01–1.5) (Table 1). Ascarid-type eggs were detected in a lion and a Bengal tiger and were morphologically consistent with members of the family Toxocaridae. Coccidian oocysts were detected in two Dall sheep, one Japanese macaque, and one elephant. Hookworm-like eggs were detected in a lion and were morphologically consistent with Ancylostomatidae, whereas trichostrongyle-type eggs were detected in a roe deer and were morphologically consistent with Trichostrongylidae.

### 3.3 Molecular detection of selected zoonotic pathogens

Among the seven selected zoonotic pathogens screened, three pathogens were detected in 14 of 367 fecal samples, corresponding to an overall molecular positivity rate of 3.8% (95% CI: 2.1–6.3) (Table 1). *E. bieneusi* was the most frequently detected pathogen (2.5%, 9/367; 95% CI: 1.1–4.6), followed by *G. intestinalis* (0.8%, 3/367; 95% CI: 0.2–2.4) and *Cryptosporidium* spp. (0.5%, 2/367; 95% CI: 0.1–2.0). *C. burnetii, T. gondii, Blastocystis* sp., and SFTSV were not detected in any samples. All *G. intestinalis*-positive samples were obtained from African crested porcupines. *Cryptosporidium* spp. was detected in two non-human primates, a southern pig-tailed macaque and a toque macaque. *E. bieneusi* was detected in a lynx, a brown bear, a hamadryas baboon, and six harbor seals. Detailed species-level sample distributions are provided in Supplementary Table S2.

### 3.4 Sequence and phylogenetic analysis of detected enteric pathogens

Sequence analysis was performed for *G. intestinalis, Cryptosporidium* spp., and *E. bieneusi* detected by molecular screening. The three *G. intestinalis* 18S rRNA sequences were identical to each other and showed 98.7–99.4% identity with assemblage B reference sequences in GenBank. In the phylogenetic tree, these sequences clustered with assemblage B sequences (Fig. 1). The two *Cryptosporidium* 18S rRNA sequences were identical to each other and showed 99.7–100% identity with reference sequences of *C. parvum*, clustering within the *C. parvum* clade (Fig. 2).

**Fig. 1:**
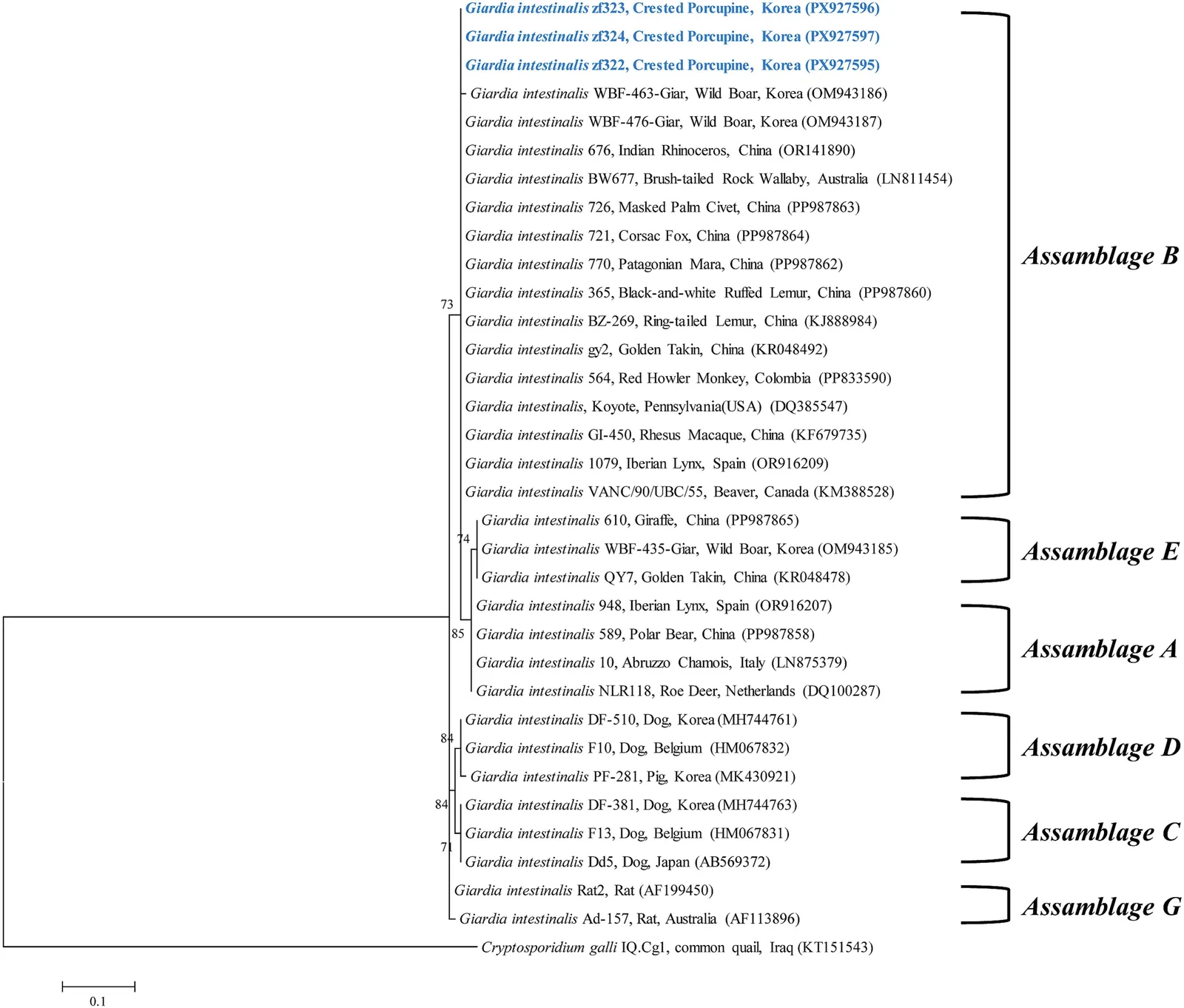
Maximum likelihood phylogenetic tree of *Giardia intestinalis* based on 18S rRNA gene sequences. Sequences from this study are highlighted in blue. *Cryptosporidium galli* was used as the outgroup. Bootstrap values from 1000 replicates are shown at the nodes. The scale bar indicates substitutions per site. (For interpretation of the references to colour in this figure legend, the reader is referred to the web version of this article.)

**Fig. 2:**
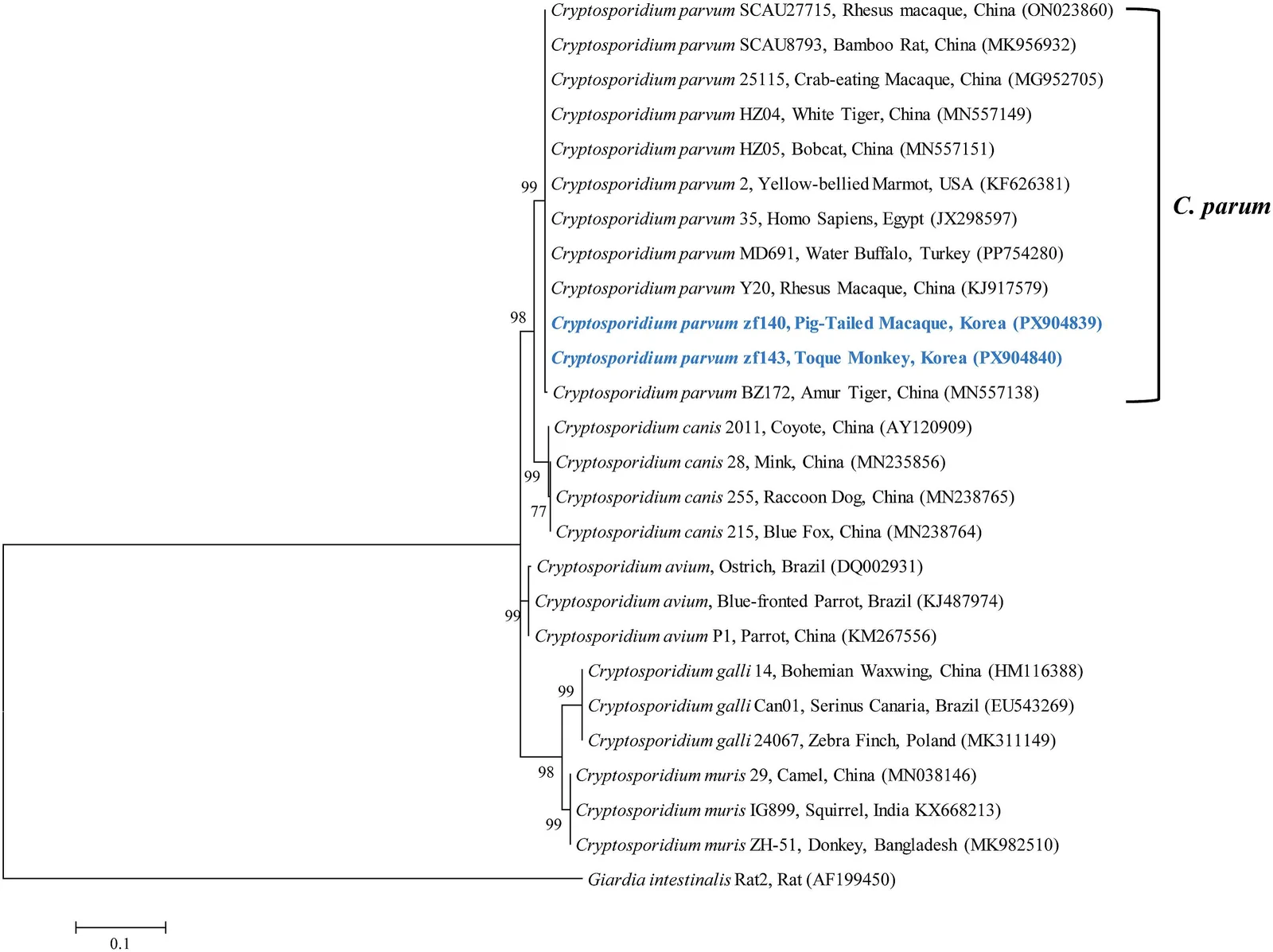
Maximum likelihood phylogenetic tree of *Cryptosporidium* spp. based on 18S rRNA gene sequences. Sequences from this study are highlighted in blue. *Giardia intestinalis* was used as the outgroup. Bootstrap values from 1000 replicates are shown at the nodes. The scale bar indicates substitutions per site. (For interpretation of the references to colour in this figure legend, the reader is referred to the web version of this article.)

For *E. bieneusi*, nine ITS sequences were obtained. Three sequences from a Eurasian lynx, a brown bear, and a hamadryas baboon were identical to each other and showed 100% identity across the complete 243-bp ITS region with the established genotype Type IV reference sequences, including KJ651436, OP851354, and OP851356. These sequences clustered within Group 1 (Fig. 3). The remaining six sequences, all obtained from harbor seals, were identical to each other but were highly divergent from previously described Group 11 reference genotypes. The closest Group 11 reference sequences included in the analysis showed approximately 90.9% ITS sequence identity. This unique ITS sequence was therefore provisionally designated as genotype SealKB1 and clustered within Group 11 (Fig. 3).

**Fig. 3:**
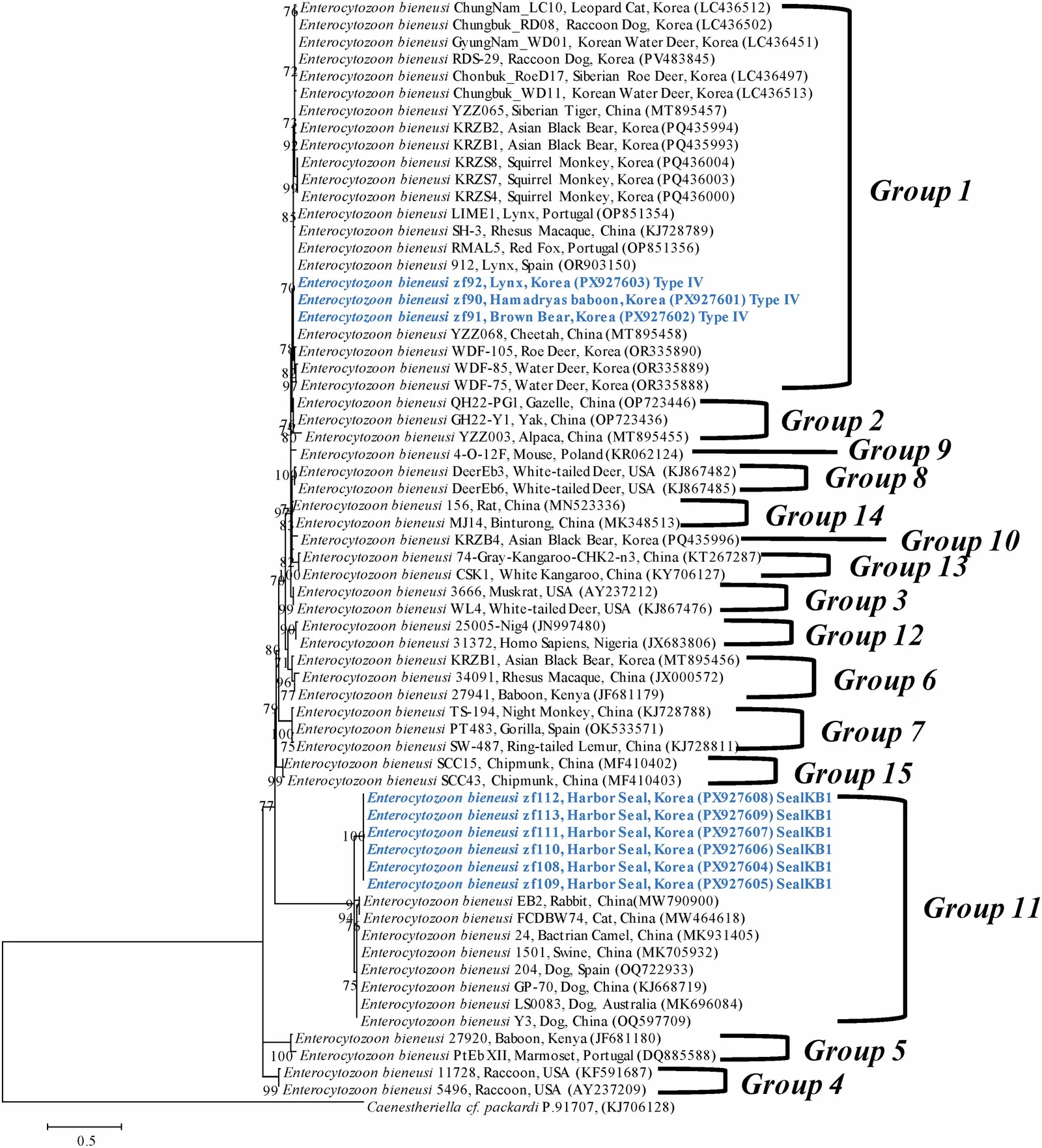
Maximum likelihood phylogenetic tree of *Enterocytozoon bieneusi* based on ITS sequences. Sequences from this study are highlighted in blue. The lynx-, brown bear-, and baboon-derived sequences were identified as genotype Type IV within Group 1, whereas the six identical harbor seal-derived sequences represented genotype SealKB1, provisionally designated as a novel genotype within Group 11. *Caenestheriella* cf. *packardi* was used as the outgroup. Bootstrap values from 1000 replicates are shown at the nodes. The scale bar indicates substitutions per site. (For interpretation of the references to colour in this figure legend, the reader is referred to the web version of this article.)

## 4 Discussion

This study investigated intestinal parasites and selected zoonotic pathogens in zoo-housed mammals in Korea using microscopic and molecular approaches. The overall positivity rate was low, which may reflect routine veterinary care, controlled feeding, enclosure hygiene, and parasite management practices in zoological institutions. Nevertheless, the detection of enteric pathogens with zoonotic potential indicates that managed zoo environments do not completely eliminate the risk of pathogen maintenance and fecal shedding. Similar molecular surveys in captive wildlife have also reported *G. intestinalis, Cryptosporidium* spp., and *E. bieneusi* in zoo-housed animals, supporting the importance of continued surveillance in zoological settings [3], [4], [5], [6], [7].

Microscopic examination detected coccidian oocysts, ascarid-type eggs, hookworm-like eggs, and trichostrongyle-type eggs at low frequencies. These findings indicate that conventional intestinal parasites may persist in zoo-housed mammals despite managed husbandry conditions. Because identification was based on morphology, the detected helminth eggs and coccidian oocysts were interpreted conservatively. Molecular characterization would be required to confirm species identity and to evaluate their pathogenic or zoonotic significance. The use of flotation alone may also have underestimated parasites that are more efficiently detected by sedimentation or other diagnostic methods.

Among the molecularly detected pathogens, *E. bieneusi* was the most frequently detected. The three identical sequences obtained from a Eurasian lynx, a brown bear, and a hamadryas baboon were identified as genotype Type IV within Group 1. Type IV is a well-established genotype reported in both humans and multiple animal hosts and is therefore considered to have zoonotic potential [8], [32]. In contrast, the six harbor seal-derived sequences represented a single distinct ITS genotype, provisionally designated SealKB1, which clustered within Group 11. The marked sequence divergence of SealKB1 from previously described Group 11 genotypes suggests that it represents a genetically distinct lineage within this group. However, because Group 11 predominantly contains host-adapted genotypes, the host range and zoonotic significance of SealKB1 remain unknown. Further investigations involving additional pinniped and marine mammal samples are warranted to clarify its host specificity, geographic distribution, and epidemiological significance.

*G. intestinalis* was detected in African crested porcupines and clustered with assemblage B reference sequences. Assemblage B has been reported in humans and various mammalian hosts, suggesting potential zoonotic relevance [9]. However, because the present analysis was based on the 18S rRNA gene, additional multilocus genotyping using loci such as *gdh, bg*, or *tpi* would be necessary for more robust assemblage-level characterization. Therefore, the present 18S rRNA data support a phylogenetic association with assemblage B but do not permit definitive assemblage assignment based on multilocus genotyping. *C. parvum* was detected in two non-human primates. This finding is epidemiologically relevant because *C. parvum* is a major zoonotic species and non-human primates may serve as potential reservoirs of human-pathogenic *Cryptosporidium* spp. [10]. However, 18S rRNA sequencing does not provide subtype-level resolution for *C. parvum*. Therefore, the gp60 subtype families of the two isolates and their specific zoonotic significance could not be determined in the present study. Additional gp60 subtyping would be required for more precise epidemiological interpretation.

*C. burnetii, T. gondii, Blastocystis* sp., and SFTSV were not detected in this study. These negative findings should not be interpreted as evidence of true absence, because detection may be influenced by host species, sampling period, pathogen shedding patterns, sample type, and assay sensitivity. Fecal sampling is appropriate for detecting intestinal organisms such as *Blastocystis*, whereas its diagnostic utility may be more limited for systemic or vector-borne pathogens. In particular, fecal samples are not the preferred specimen for detecting SFTSV, and blood or tick samples would be more appropriate for assessing SFTSV infection or exposure. Similarly, detection of *C. burnetii* and *T. gondii* in feces may depend strongly on host species and shedding dynamics. Therefore, the absence of molecular detection in the present fecal samples should not be interpreted as evidence that these pathogens are absent from zoo-housed mammals. Future surveillance incorporating pathogen-appropriate specimens and repeated or seasonally distributed sampling would provide a more comprehensive assessment.

From a One Health perspective, the detection of enteric pathogens in zoo-housed mammals is relevant to animal health, environmental hygiene, occupational exposure, and public health risk assessment. Fecal shedding by captive wildlife may contribute to contamination of enclosures, bedding materials, water sources, and cleaning equipment, potentially facilitating repeated exposure among animals and indirect exposure of zookeepers, veterinarians, and visitors. In particular, personnel involved in daily husbandry, fecal removal, veterinary procedures, and enclosure cleaning may have greater opportunities for exposure to contaminated fecal material and environmental surfaces. Appropriate use of personal protective equipment, hand hygiene, dedicated cleaning equipment, and routine disinfection should therefore be emphasized, particularly in enclosures housing animals positive for zoonotic enteric pathogens. Although this study did not include human or environmental samples, environmental sampling of water, soil, bedding, and frequently contacted surfaces could complement animal-based surveillance by identifying potential contamination pathways. In addition, visitor-facing areas should be included in biosecurity assessments, particularly where direct or indirect contact with animals or contaminated environments is possible. These recommendations are also consistent with the current regulatory framework for zoological institutions in Korea. Under the Act on the Management of Zoos and Aquariums and its Enforcement Decree, zoo operators are required to conduct regular health monitoring of captive animals, including periodic examinations by veterinarians, for which fecal examination is one of the specified diagnostic approaches [33]. Our findings suggest that molecular pathogen screening and targeted environmental monitoring could complement these existing animal health management requirements and strengthen One Health-oriented biosecurity in Korean zoological institutions.

This study has several limitations. First, the cross-sectional design and limited sampling period may not reflect seasonal variation in pathogen shedding. Second, only fecal samples were analyzed, and human, tick, and environmental samples were not included. Third, information on clinical signs, recent antiparasitic treatment, enclosure type, and management practices was not available for all animals. **Fourth, sample sizes were highly uneven among host taxonomic groups, with several groups represented by only a small number of animals. Therefore, subgroup-specific positivity estimates may be unstable and should not be interpreted as evidence of differences in infection risk among host taxa.** Fifth, microscopic examination and single-locus molecular analyses may have limited diagnostic, genotyping, and subtype resolution. Finally, some pathogens included in the molecular panel may require different sample types for optimal detection. These limitations should be considered when interpreting the prevalence estimates and One Health implications of the findings.

## 5 Conclusions

This study provides baseline data on intestinal parasites and selected zoonotic pathogens in zoo-housed mammals in Korea. Although the overall positivity rate was low, the detection of *E. bieneusi, G. intestinalis*, and *C. parvum* indicates that captive zoo mammals can harbor enteric pathogens of potential zoonotic relevance. These findings support the need for continued fecal surveillance, molecular characterization, and appropriate biosecurity practices in zoological institutions. Future studies incorporating animal, environmental, and human-interface samples will be important for strengthening One Health-based risk assessment and pathogen management in zoo settings.

## CRediT authorship contribution statement

**Hyo-Min Woo:** Writing – original draft, Methodology, Investigation, Formal analysis. **In Jung Jung:** Writing – original draft, Methodology, Investigation, Formal analysis. **Beoul Kim:** Software, Resources, Investigation. **You-Jeong Lee:** Visualization, Validation, Formal analysis. **Jae-Woo Choi:** Visualization, Methodology, Formal analysis. **Insu Choi:** Software, Resources, Investigation. **Garam Kim:** Visualization, Resources, Methodology, Data curation. **Weon-Hwa Jheong:** Validation, Supervision, Resources, Data curation. **Byoungcheun Lee:** Validation, Supervision, Project administration, Funding acquisition. **Kyoo-Tae Kim:** Writing – review & editing, Writing – original draft, Supervision, Funding acquisition, Conceptualization. **Min-Goo Seo:** Writing – review & editing, Writing – original draft, Funding acquisition, Conceptualization.

## Ethics declaration

This study was conducted in accordance with the ARRIVE (Animal Research: Reporting of In Vivo Experiments) guidelines. This study was approved by the Institutional Animal Care and Use Committee of Kyungpook National University. (Approval No. 2025–0902)

## Funding

This work was supported by a grant from the National Institute of Wildlife Disease Control and Prevention (NIWDC), funded by the Ministry of Climate, Energy and Environment of the Republic of Korea (NIWDC-2024-SP-06).

## Declaration of competing interest

The authors declare that they have no known competing financial interests or personal relationships that could have appeared to influence the work reported in this paper.

## Data Availability

The data supporting the findings of this study are included within the article.
