# Supplementary material for: Prevalence and molecular characterization of enteric zoonotic pathogens in zoo-housed mammals in Korea
Source: One Health. 2026 Sep 23;23:101568. doi: 10.1016/j.onehlt.2026.101568 (PMC13636016; doi:10.1016/j.onehlt.2026.101568)
Supplement: Supplementary file 1 [file mmc1.docx]

| **Pathogen** | **Target gene/region** | **PCR type** | **Primer set** | **Amplicon size** | **Reference** |
| --- | --- | --- | --- | --- | --- |
| *Coxiella burnetii* | IS1111 | Conventional PCR | Trans1/Trans2 | 687 bp | [23] |
| *Toxoplasma gondii* | B1 | Nested PCR | S1/AS1; S2/AS2 | 530 bp | [24] |
| *Giardia intestinalis* | 18S rRNA | Nested PCR | RH11/RH4LM; GiarF/GiarR | 174 bp | [25] |
| *Cryptosporidium* spp. | 18S rRNA | Nested PCR | 18SiCF2/18SiCR2; 18SiCF1/18SiCR1 | 587 bp | [26] |
| *Enterocytozoon bieneusi* | ITS | Nested PCR | ITSF1/ITSR1; ITSF2/ITSR2 | 389 bp | [27] |
| *Blastocystis* sp. | 18S rRNA | PCR | RD5/BhRDr | 600 bp | [28] |
| SFTSV | S segment | Nested RT-PCR | Np2F/Np2R; Nn2F/Nn2R | 346 bp | [29] |

**Supplementary Table S1.** Primers and PCR conditions used for molecular detection of selected zoonotic pathogens.
